# New Perspectives on Antimicrobial Agents: Omadacycline for community-acquired pneumonia, skin and soft tissue infections, and nontuberculous mycobacteria (focus on *M. abscessus*)

**DOI:** 10.1128/aac.01087-24

**Published:** 2025-01-16

**Authors:** Thomas M. File, Julio A. Ramirez, Ashley M. Wilde

**Affiliations:** 1 Division of Infectious Disease, Summa Health 1080 , Akron, Ohio, USA; 2 Section of Infectious Diseases, Northeast Ohio Medical University 6969 , Rootstown, Ohio, USA; 3 Division of Infectious Diseases, University of Louisville 277605 , Louisville, Kentucky, USA; 4 Norton Infectious Diseases Institute, Norton Healthcare 5166 , Louisville, Kentucky, USA; University of Pittsburgh School of Medicine, Pittsburgh, Pennsylvania, USA

**Keywords:** omadacycline, pneumonia, skin infections, clinical use

## Abstract

Omadacycline is a novel antimicrobial belonging to the tetracycline class. It has the ability to evade both efflux and ribosomal methylation types of resistance and therefore has an expanded spectrum compared to other tetracycline agents. Omadacycline is active against a number of multidrug-resistant bacteria, including macrolide and doxycycline-resistant *Streptococcus pneumoniae,* methicillin-resistant *Staphylococcus aureus* (MRSA), vancomycin-resistant Enterococcus, and several enteric gram-negative bacilli. It also has activity against many nontuberculous mycobacterium (NTM) species. It is available both orally and intravenously, which allows for feasible switch therapy. This review will assess the antimicrobial activity, pharmacology, safety, and clinical efficacy of omadacycline and present the opinions of the authors on where to position omadacycline for clinical practice.

## PERSPECTIVE

Omadacycline is a semisynthetic aminomethylcycline antibiotic derived from minocycline. It is approved for both community-acquired bacterial pneumonia (CABP) and acute bacterial skin and skin structure infections (ABSSSI) in the United States and China and has orphan indication as a first-line agent for nontuberculous mycobacteria (NTM) in the United States (1, 2).

## MECHANISM OF ACTION

Omadacycline inhibits bacterial protein synthesis by binding the 30S ribosomal subunit at the tetracycline-binding site. Through modifications at the C-9 position with an aminomethyl group on the tetracycline D-ring, omadacycline is protected against the two major tetracycline resistance determinants: ribosomal protection and drug efflux (3). Ribosomal protection is mediated by proteins that block the binding of tetracyclines such as doxycycline, but not omadacycline, to the ribosome. Omadacycline has demonstrated low MICs *in vitro* against bacteria carrying ribosomal protection [tet(M), tet(O), tet(S)] and efflux [tet(K), tet(L), tet(A), and tet(B)] resistance genes (4, 5). Inducible mechanisms of omadacycline resistance have not been identified (6). The C-9 modification of omadacycline was designed to improve the bioavailability and side effect profile, specifically nausea, of its predecessor tigecycline (7). Omadacycline has broad immunomodulatory activity. In an *in vitro* study, omadacycline reduced TNF-α, IL-1β, IL-6, IL-4, IL-10, and IFN-γ responses when human monocyte cells were exposed to *Escherichia coli* lipopolysaccharide (8).

## PHARMACOKINETICS/PHARMACODYNAMICS

Omadacycline is available both as an oral or intravenous formulation. The bioavailability of oral omadacycline is 34.5% following a single dose, leading to the recommended maintenance oral dose of 300 mg compared to the IV dose of 100 mg (1). Like other tetracyclines, omadacycline absorption is impacted by the presence of food and chelated when administered with multivalent cations such as aluminum-, calcium-, magnesium-, iron-, and bismuth-containing products. Because of the impact of food and multivalent cations, it is recommended in the prescribing information that patients taking oral omadacycline should fast for at least 4 hours and then take with water. After oral dosing, no food or drink (except water) is to be consumed for 2 hours and no dairy products, antacids, or multivitamins for 4 hours.

Omadacycline has a high apparent volume of distribution and penetrates well into the epithelial lining fluid and alveolar cells (1, 9). Omadacycline is primarily eliminated in the feces (81.1%) and urine (14.4%). Omadacycline is not a substrate, inducer, or inhibitor of nine tested cytochrome P450 enzymes or five other known drug transporters. No adjustments in dose are recommended based on patient age, sex, body mass index (BMI), comorbidities, hepatic function, or renal function. In a study of patients on hemodialysis, only 7.89% of omadacycline was recovered from the dialysate of patients administered omadacycline before hemodialysis, suggesting that omadacycline can be administered irrespective of the dialysis schedule (10).

Due to its long half-life, omadacycline was studied using an IV or oral loading strategy to administer 300 mg IV or 900 mg orally over the first 2 days to optimize pharmacodynamic exposure early in the treatment course. Oral loading dose strategies differ between Food and Drug Administration (FDA)-approved indications (1). While the effects of 450 mg orally once daily on days 1 and 2 were studied in a clinical trial for ABSSI, the recommendation for CAP is 300 mg two doses on day 1, which is based on a pharmacokinetic study demonstrating omadacycline exposures similar to the IV loading dose used in the CAP clinical trial (11). Full details of omadacycline dosing recommendations can be found in Table 1 (1).

**TABLE 1:** Omadacycline dosing recommendations^*a*^

Indication	Loading dose options		Maintenance dose
Acute bacterial skin and skin structure infections	IV^*b*^: Day 1, 200 mg once OR 100 mg twice Oral: Days 1 and 2, 450 mg daily		IV: 100 mg daily Oral: 300 mg daily
Community-acquired bacterial pneumonia	IV: Day 1, 200 mg once or 100 mg twice Oral: Day 1, 300 mg twice		IV: 100 mg daily Oral: 300 mg daily

^*a*^ From reference 1.

^*b*^ IV, intravenous.

## BACTERIAL SUSCEPTIBILITY

Like all tetracyclines, omadacycline exhibits broad-spectrum activity against aerobic and anaerobic gram-positive and gram-negative bacteria as well as atypical pathogens. Omadacycline notably lacks activity against *Proteus* spp., *Providencia* spp., *Pseudomonas* spp., *Morganella* spp., and *Eikenella corrodens* with MIC_90_ ≥16 mcg/mL (12, 13). Currently, only FDA breakpoints are available for omadacycline (14). Activities of omadacycline against contemporary isolates from the United States are summarized in Table 2. Interest in omadacycline for other organisms has increased due to its potential clinical efficacy in unusual or difficult-to-treat bacterial infections. Omadacycline holds promise for the treatment of nontuberculous mycobacterium (15) (discussed in greater detail below). Omadacycline minimum inhibitory concentrations (MICs) against multi-drug resistant (MDR) gram-negative bacteria vary. MIC data of common MDR pathogens as well as additional uncommon pathogens are summarized in Table 3. Like doxycycline, omadacycline demonstrates *in vitro* activity against anaerobes including *Clostridioides difficile*, which may decrease the risk of *C. difficile* infections (16) (discussed in the “Safety” section below).

**TABLE 2:** *In vitro* activity of omadacycline in the United States in 2020–21^*a*^

Organism	Number of isolates	MIC_50_ (mcg/mL)	MIC_90_ (mcg/mL)	% Susceptible
Skin and skin structure infections				
*Staphylococcus aureus*	1,575	0.12	0.12	99.0
MRSA	659	0.12	0.25	98.0
MSSA	916	0.12	0.12	99.8
*Staphylococcus lugdunensis*	41	0.06	0.06	97.6
*Streptococcus anginosus* group	22	0.06	0.06	95.5
*Streptococcus pyogenes*	104	0.06	0.12	100
*S. pyogenes* macrolide-R	28	0.06	0.12	100
*S. pyogenes* tetracycline-R	35	0.06	0.12	100
*Enterococcus faecalis*	114	0.06	0.12	100
*E. faecalis* vancomycin-R	4	0.12	–^*b*^	100
*Enterococcus faecium*	30	0.06	0.12	Unavailable
*E. faecium* vancomycin-R	21	0.06	0.12	Unavailable
*Enterobacter cloacae* species complex	88	2	8	87.5
*Klebsiella pneumoniae*	99	2	8	88.9
*Escherichia coli*	174	0.5	2	Unavailable
Community-acquired respiratory tract infections				
*Streptococcus pneumoniae*	594	0.06	0.06	99.8
*S. pneumoniae* penicillin-R	64	0.06	0.06	100
*S. pneumoniae* macrolide-R	270	0.06	0.06	100
*S. pneumoniae* tetracycline-R	119	0.06	0.06	100
*Haemophilus influenzae*	371	0.5	1	99.7
*Moraxella catarrhalis*	193	≤0.12	0.25	Unavailable
Pneumonia in hospitalized patients				
*Staphylococcus aureus*	832	0.12	0.25	Unavailable
MRSA	321	0.12	0.5	Unavailable
MSSA	511	0.12	0.12	99.6
*Streptococcus pneumoniae*	12	0.06	0.06	100
*Haemophilus influenzae*	32	0.5	1	100
*Acinetobacter baumannii–calcoaceticus* species complex	110	0.5	4	90.0^*c*^
*Klebsiella pneumoniae*	281	2	8	88.3
*Escherichia coli*	214	0.5	2	99.1^*c*^

^*a*^ Source: Huband et al. (17).

^*b*^ –, not reported.

^*c*^ Percent inhibited at ≤4 mcg/mL.

**TABLE 3:** *In vitro* activity of omadacycline against uncommon pathogens

Organism (reference)	Number of isolates	MIC_50_ (mcg/mL)	MIC_90_ (mcg/mL)	MIC range (mcg/mL)
Gram-positive organisms				
*Bacillus anthracis* (18)	53	0.015	0.03	<0.008 to 0.25
*Clostridioides difficile* (16)	200	0.0625	0.125	0.031 to 0.25
*Corynebacterium* spp. (19)	10	0.12	0.5	–^*a*^
*Listeria monocytogenes* (19)	10	0.12	0.12	–
*Nocardia* spp. (20)	300	2	4	0.06 to 8
*N. abscessus* complex	16	0.5	1	0.06 to 2
*N. asiatica*	10	0.25	1	0.06 to 1
*N. beijingensis*	10	0.5	2	0.12 to 4
*N. brasiliencsis*	19	2	2	0.25 to 2
*N. cyriacigeorgica*	64	2	4	0.5 to 4
*N. farcinica*	36	4	8	two to 8
*N. nova*	59	4	4	0.5 to 8
*N. otitidiscaviarum*	11	1	2	0.25 to 2
*N. paucivorans*	11	0.25	0.25	0.12 to 0.25
*N. veterana*	14	4	4	2 to 4
*N. wallacei*	12	4	4	0.25 to 8
Other *Nocardia* spp.	38	2	4	0.06 to 8
Gram-negative organisms				
*Achromobacter xylosoxidans* (19)	10	2	8	–
*Acinetobacter baumannii*				
Bloodstream isolates: Taiwan (21)	255	4	8	0.12 to 32
Bloodstream isolates: Greece (22)	271	8	>32	0.12 to >32
Carbapenem-nonsusceptible isolates (23)	41	4	8	1 to 16
*Aeromonas* spp. (19)	10	1	1	–
*Alcaligenes faecalis* (19)	10	4	8	–
*Burkholderia cepacia* species complex (19)	10	1	4	–
Enterobacterales – MDR^*b*^ (24)				
*Escherichia coli*: ESBL phenotype	1,784	1	2	0.12 to 32
*Escherichia coli*: carbapenem-resistant	13	1	2	0.5 to 4
*Klebsiella pneumoniae*: ESBL phenotype	1,383	4	8	0.25 to >32
*Klebsiella pneumoniae*: carbapenem resistant	388	4	8	0.5 to >32
*Enterobacter cloacae* species complex: ceftazidime-nonsusceptible	478	2	8	0.25 to 32
*Stenotrophomonas maltophilia* (25)	41	8	32	0.5 to >64
*Yersinia pestis* (26)	30	1	1	0.12 to 2
Other organisms				
Rapidly growing mycobacteria^*c*^ (27)				
*M. abscessus* subsp. *abscessus*	20	0.12	0.25	0.06 to 0.5
*M. abscessus* subsp. *massiliense*	3	0.12	–	0.06 to 0.25
*M. chelonae*	15	0.12	0.5	0.03 to 0.5
*M. fortuitum* group	12	0.12	0.25	0.06 to 0.25
Doxycycline nonsusceptible^*d*^	5	0.12	0.25	0.06 to 0.25
Doxycycline susceptible	7	0.12	0.25	0.12 to 0.25
*M. goodii*	2	–	–	0.06
*M. immunogenum*	7	0.25	0.5	0.03 to 0.5
*M. mucogenicum* group	10	0.25	0.5	0.12 to 1
Doxycycline nonsusceptible	4	0.12	–	0.12 to 0.5
Doxycycline susceptible	6	0.25	1	0.25 to 1
*M. wolinskyi*	1	–	–	0.5
Slowly growing mycobacteria^*e*^ (27)				
*M. arupense*	6	>16	>16	1 to >16
*M. avium* complex	16	>16	>16	0.06 to >16
*M. kansasii*	5	8	16	4 to >16
*M. lentiflavum*	3	>16	–	4 to >16
*M. marinum*	2	–	–	4 to 16
*M. paraffinicum*	2	–	–	8 to >16
*M. simiae*	7	>16	>16	>16
*Mycoplasma genitalium* (28)	10	0.125	0.25	0.063 to 0.5

^*a*^ –, not reported.

^*b*^ MDR, multidrug resistant.

^*c*^ MICs described are at 100% inhibition.

^*d*^ Doxycycline nonsusceptible defined as MICs ≥2 mcg/mL.

^*e*^ Without supplemental omadacycline added to media; may be artificially elevated due to omadacycline degradation in media.

## SAFETY

The safety profile of omadacycline was evaluated in a review of the adverse event profiles of omadacycline in phase 3 clinical trials for acute bacterial skin and skin structural infections (ABSSSI; comparator linezolid) and community-acquired pneumonia (CAP; comparator moxifloxacin) (29). Treatment-emergent AEs occurred in similar percentages of omadacycline (47.5%), linezolid (41.2%), and moxifloxacin (48.5%) patients. Serious AEs occurred in 3.6% of omadacycline patients, 1.9% of linezolid patients, and 6.7% of moxifloxacin patients. Overall mortality in the compilation of the three clinical trials was comparable. In the Omadacycline for Pneumonia Treatment In the Community (OPTIC) 1 trial, there was a slight imbalance with eight vs four deaths in the omadacycline arm vs the moxifloxacin arm. However, in the OPTIC 2 study (discussed below and which enrolled only patients with Patient Outcomes Research Team (PORT) III or IV severity), the mortality was balanced with six deaths in each arm (30).

Gastrointestinal AEs were the most frequent events in all treatment groups. Nausea (14.9% omadacycline, 8.7% linezolid, and 5.4% moxifloxacin) and vomiting (8.3% omadacycline, 3.9% linezolid, and 1.5% moxifloxacin) were the most frequently reported. Most of the gastrointestinal upset incidences in the omadacycline arm were associated with oral administration and particularly with the 450 mg loading dose during the first 2 days of the OASIS-2 study during which nausea and vomiting occurred in 25.3% and 12.5% of patients receiving omadacycline, respectively. After day 2 through end of treatment, onset of nausea and vomiting each occurred in 4.1% of omadacycline patients.

Preclinical studies have shown that omadacycline inhibits carbamylcholine binding to the M2 subtype of the muscarinic acetylcholine receptor, resulting in a transient, generally asymptomatic increase in heart rate (31). In the clinical trials, the increase rate from baseline to post-dose was approximately five beats/minute. Omadacycline has minimal effect on the QTc interval.

### Risk of *Clostridioides difficile* infection

Several studies have shown high levels of *in vitro* activity of omadacycline against *C. difficile* (32–34). In a study evaluating the activity of omadacycline and five comparators using agar dilution assay, the MIC_90_s were as follows: Omadacycline 1 mcg/mL; metronidazole 1 mcg/mL; vancomycin 2 mcg/mL; fidaxomicin 0.5 mcg/mL; tigecycline 0.12 mcg/mL (16). Because approximately 80% of omadacycline is present in the stool, this implies a high level of antimicrobial effect. In a study which evaluated the extent of *C. difficile* spore eradication when germinants were combined with omadacycline or vancomycin, there was >99% spore eradication with omadacycline and >94% for vancomycin (35). Exposure of omadacycline in an *in vitro* model that simulates the human colon did not induce *C. difficile* proliferation or toxin production; and omadacycline did not cause modeled CDI (36). In a hamster model of CDI used for assessing survival, omadacycline performed better than vancomycin (37). In the clinical trials, no patients in the omadacycline arms experienced CDI, whereas 2.1% of patients in the moxifloxacin arm of the OPTIC-1 trial developed CDI (38).

## CLINICAL STUDIES ON COMMUNITY-ACQUIRED BACTERIAL PNEUMONIA (CABP) AND ACUTE BACTERIAL SKIN AND SKIN STRUCTURE INFECTIONS (ABSSSI)

The OPTIC trial compared omadacycline with moxifloxacin in 774 patients with CABP. Results demonstrated the noninferiority of omadacycline, with early clinical response (ECR) rates of 81% vs 83% for moxifloxacin and post-treatment evaluation (PTE) response rates of 88% vs 85% (38). This established omadacycline as a viable alternative for CABP, with similar efficacy and safety profiles and a benefit against drug-resistant pathogens.

In additional OPTIC data analyses, ECR—defined as symptom improvement within 72–120 hours—was shown to be a reliable endpoint for evaluating CABP treatment, strongly correlating with clinical stability (vital sign normalization) and predicting positive PTE outcomes (39). Patients reaching clinical stability within the ECR window generally showed continued improvement, highlighting ECR as a practical tool for monitoring CABP.

Secondary OPTIC analyses examined omadacycline’s efficacy in patients with varying CABP severity, comorbid conditions (e.g., COPD, asthma, and diabetes), and radiographic features. Across these variables, omadacycline’s clinical success was comparable to that of moxifloxacin (40). A specific subgroup analysis indicated slightly lower success in bacteremia cases for omadacycline, but overall, it proved effective regardless of disease severity or risk factors. Additional studies according to European Medicines Agency guidelines confirmed omadacycline’s efficacy in higher-risk populations (PORT risk class III and IV), with high clinical success rates similar to those of moxifloxacin (41).

A subsequent OPTIC analysis of patients with comorbidities found high ECR rates in both omadacycline (92%) and moxifloxacin (91%) groups. Even among patients with multiple comorbidities, response rates remained high, showing omadacycline’s effectiveness across various health profiles. Safety profiles were similar, with adverse event rates low for both drugs (42).

Preliminary results from a second phase 3 randomized CABP trial (OPTIC-2) released in 2024 compared omadacycline with moxifloxacin in 670 adults with moderate-to-severe CABP (30). Clinical success rates were high in both groups (ECR rates: 89.6% for omadacycline vs 87.7% for moxifloxacin). Post-treatment success was 86.0% for omadacycline and 87.7% for moxifloxacin, consistent across patient risk classes and pathogen types. Omadacycline was well-tolerated, with comparable safety outcomes: treatment-emergent adverse events (TEAEs) were 27.7% for omadacycline vs 23.5% for moxifloxacin. The overall mortality rate was 1.8% and balanced with six deaths in each treatment arm. This trial reinforced omadacycline’s role as an alternative treatment option for CABP.

Omadacycline was evaluated in the Omadacycline for Acute bacterial Skin and Skin-Structure Infections (OASIS-1 and OASIS-2) trials for treating ABSSSIs, which include cellulitis, erysipelas, wound infections, and major abscesses. In OASIS-1, omadacycline was compared with linezolid, with both drugs showing similar early clinical response rates (85% for omadacycline vs 86% for linezolid), achieving noninferiority (43). OASIS-2, which involved oral-only administration, showed 88% ECR for omadacycline vs 83% for linezolid (44). Across different infection types, omadacycline matched linezolid’s efficacy, including against methicillin-resistant *Staphylococcus aureus* (MRSA). In the OASIS 2 trial, the clinical response rate to omadacycline among 104 patients with MRSA was 86%, compared to 79% among 107 patients treated with linezolid. Similarly, in the OASIS 1 trial, the clinical response rate to omadacycline among 69 patients with MRSA was 83%, compared to 86% among 50 patients treated with linezolid.

Further analysis combined OASIS-1 and OASIS-2 data to explore omadacycline’s effectiveness in persons who inject drugs (PWID), showing comparable success rates to those obtained with linezolid (over 90% at PTE) (45). Another analysis addressed omadacycline’s efficacy across BMI categories and in diabetic patients. Omadacycline’s success was consistent across BMI categories, with no significant reduction in efficacy in higher BMI classes. Diabetic patients had slightly lower ECR rates, but outcomes at PTE were similar for omadacycline and linezolid, indicating no need for dose adjustments based on BMI or diabetes status (46).

A combined data set from OPTIC-1, OASIS-1, and OASIS-2 assessed the efficacy of omadacycline in patients with secondary bacteremia and those with renal impairment. The secondary bacteremia study evaluated a total of 63 patients, 30 patients with ABSSSI and 33 patients with CABP (47). Omadacycline achieved ECR in 76% and clinical success in 79% at PTE, treating gram-positive infections effectively, including MRSA and *Streptococcus* species (47). In CABP cases, omadacycline was also effective against pathogens like *Streptococcus pneumoniae* and *Klebsiella pneumoniae*. Among patients with CABP bacteremia, the rate of clinical response at PTE was 73% (11 of 15) with omadacycline and 83% (15 of 18) with moxifloxacin. Investigators attributed these differences to the small sample size and indeterminate responses. In patients with mild-to-moderate renal impairment, omadacycline demonstrated high success rates across all renal function groups without requiring dosage adjustments, performing similarly to moxifloxacin for CABP and linezolid for ABSSSI (48).

## CLINICAL STUDIES FOR NONTUBERCULOUS MYCOBACTERIA (NTM)

NTM has been observed to be an increasing contributor to clinical infections over the past couple of decades. Of these, the rapid growers (*M. abscessus, M. chelonae, and M. fortuitum*) are difficult to treat due to resistance to commonly used antimicrobials, including macrolides. Newer agents are therefore sought for therapy.

Several studies have evaluated the *in vitro* activity of omadacycline against isolates of NTM and demonstrated high level of potency (49–51). This includes activity against strains that are resistant to many antimicrobial agents used to treat NTM, including clarithromycin, azithromycin, amikacin, cefoxitin, and imipenem. In addition, synergy studies have shown the potentiation of the combination of omadacycline with clarithromycin (50).

The evidence for clinical efficacy of omadacycline for NTM treatment is primarily based on case reports and case series of real-world experience and mostly for *M. abscessus,* for which the majority report a positive response (52–55).

In most cases, omadacycline was part of a combination antimicrobial regimen and often administered as part of an oral therapy regimen after an initial intravenous therapy regimen. However, in one study by Duah et al*.*, omadacycline was used as part of first-line treatment regimen for patients with *M. abscessus* pulmonary infection (55). Often omadacycline was included in the treatment regimen due to progression of diseases or due to intolerability to components of the initial regimen.

Two multicenter retrospective studies have assessed the long-term effect of omadacycline in the treatment of NTM infections. In a study across 16 U.S. medical institutions, El Ghali et al. examined the long-term clinical success, safety, and tolerability of omadacycline for 75 NTM infections (15). Most had NTM pulmonary disease (33/75, 44.0%), skin and soft tissue disease (19/75, 25.3%), or osteomyelitis (10/75, 13.3%), and *M. abscessus* (60/75, 80%) was the most commonly isolated NTM pathogen. The median treatment duration was 6 months (longest 14 months), and the most commonly co-administered antibiotic was azithromycin (33/70, 47.1%). Three-month clinical success was observed in 80.0% (60/75) of patients, and AEs attributable to omadacycline occurred in 32.0% (88% of these were gastrointestinal) of patients, leading to drug discontinuation in 9.3% (7/75). In another multicenter retrospective chart review of adults with *M. abscessus* infection (most had lung infection), 117 patients were evaluated for long-term safety and tolerability (56). The median duration of omadacycline treatment was 8 months. Twenty-three experienced intolerance or adverse events that led to drug cessation. In those with pulmonary disease, 44 of 95 (46%) had one or more negative cultures at the time of final microbiological assessment, with 17 of 95 (18%) achieving culture conversion. The authors concluded the data supported long-term safety and tolerability of omadacycline along with potential effectiveness in treatment of *M. abscessus* infections.

## CONSIDERATIONS FOR USE IN CLINICAL PRACTICE

Because of the *in vitro* activity, as well as the safety and pharmacokinetic profiles, omadacycline offers several considerations for clinical practice.

### Community-acquired pneumonia (CAP)

For CAP, omadacycline is active against the key pathogens including *S. pneumoniae*, *H. influenzae,* and organisms associated with atypical pneumonia. Particularly, it is active against doxycycline- and macrolide-resistant *S. pneumoniae* that occurs in approximately 20%–40% of isolates from North America. It also displays *in vitro* activity against many Enterobacterales (e.g., *K. pneumoniae* and *E. coli*) and MRSA, which may be causes of CAP in selected patients.

**Fig 1:**
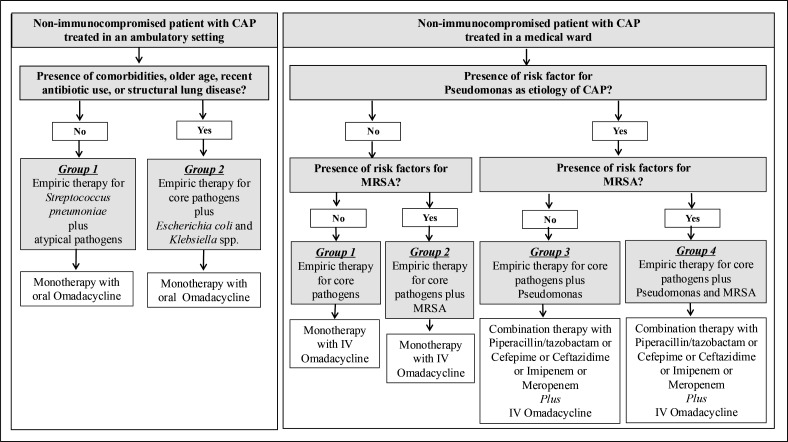
Algorithm for use of omadacycline for bacterial causes of CAP.

Our approach for use of omadacycline in the treatment of CAP is shown in Fig. 1 (57–59). Empiric antibiotic therapy targets the likely bacteria based on the presence of specific risk factors. For ambulatory non-immunocompromised patients who are healthy, under 65 years of age, and have no recent history of antibiotic use, empiric antibiotic therapy targets *S. pneumoniae* and atypical pathogens such as *Mycoplasma pneumoniae*, *Legionella pneumophila*, and *Chlamydia pneumoniae*) (Fig. 1: ambulatory setting Group 1). For ambulatory non-immunocompromised patients with medical comorbidities, older age, recent antibiotic use, or who smoke or have alcohol use disorder (Fig. 1: ambulatory setting Group 2), empiric antibiotic therapy should expand coverage to include other core pathogens such as *Haemophilus influenzae*, *Moraxella catarrhalis*, and methicillin-susceptible *S. aureus* (MSSA), as well as Enterobacterales (e.g., *E. coli* and *Klebsiella* spp.). Patients in both Groups 1 and 2 receiving CAP treatment in the ambulatory setting (Fig. 1) can be managed with oral omadacycline with a loading dose of 600 mg on day 1 divided into two doses, followed by 300 mg daily.

Hospitalized non-immunocompromised patients admitted to the medical ward are categorized into four groups based on the presence of risk factors for *Pseudomonas aeruginosa* or MRSA. Strong risk factors for *P. aeruginosa* include known colonization or previous infection and gram-negative bacilli on Gram stain. Strong risk factors for MRSA include known colonization or previous infection and gram-positive cocci in clusters on Gram stain. Patients without risk factors for *Pseudomonas* or MRSA (Fig. 1: medical ward Group 1) or patients without risk factors for *Pseudomonas* but risk factors for MRSA (Fig. 1: medical ward Group 2) can be managed with intravenous omadacycline starting with a loading dose on day 1 of 200 mg, followed by 100 mg daily. Once patients reach clinical stability, intravenous omadacycline can be switched to oral therapy at a dose of 300 mg daily. Patients with risk factors for *P. aeruginosa* with or without risk factors for MRSA (Fig. 1: medical ward Groups 3 and 4) can be managed with intravenous omadacycline as part of a combination therapy including an antipseudomonal intravenous antibiotic.

Although not indicated by the FDA for MRSA CAP, omadacycline has activity against MRSA and may provide an extra level of confidence if there is a possibility of MRSA. The results of the recently completed OPTIC 2 study for seriously ill patients (only PORT III or IV) provide evidence for use of omadacycline as monotherapy. This supports its use for patients admitted to the general ward in place of a respiratory fluoroquinolone for which there is potential for significant adverse effects. Presently, there is limited evidence assessing its use for patients with severe CAP who are admitted to the ICU.

The clinical trials of omadacycline for CAP were conducted when the recommended duration of therapy was 7 to 14 days. However, the current recommendation is a shorter course of 5 days for patients who demonstrate early clinical improvement (57). We believe that patients with CAP who achieve clinical stability within 3 days of omadacycline therapy can be safely treated with a 5-day course.

Omadacycline does not prolong the QTc interval and is much less likely to induce CDI as compared to ceftriaxone, a macrolide or a fluoroquinolone as described above. In order to assess the relative difference between omadacycline and a fluoroquinolone related to the risk of CDI, Lodise et al*.* developed a conceptual healthcare-decision analytic model to estimate incremental costs associated with treating the inpatients with a regimen of omadacycline instead of moxifloxacin (60). In this model, the incidence of excess CDI in moxifloxacin-treated patients would need to be 5%–10% for omadacycline to be cost-saving assuming the attributable CDI cost is approximately $30,000. They concluded targeted omadacycline use may reduce economic burden associated with hospitalized CABP patients due to associated CDI risk. Thus, omadacycline can be an optimal choice for treatment of CAP in patients who are at high risk of CDI, especially those with multiple comorbidities, advanced age, significant healthcare or high-risk antibiotic exposure, or who have had CDI in the past. Additionally, as omadacycline does have some immunomodulatory activity (8), this may provide a “macrolide-like-benefit” to the host response.

### Acute bacterial skin and soft tissue infections (ABSSSI)

The MRSA activity of omadacycline is relevant for ABSSSI, for which the drug has approved indications for both MSSA and MRSA. It also has good activity for *S. pyogenes* and other relevant gram-positive cocci skin pathogens (e.g., *S. lugdunensis*, *Streptococcus anginosus* group. including *S. anginosus*, *S. intermedius*, and *S. constellatus*). In addition, omadacycline has activity against *Enterococcus faecalis* and many gram-negative bacilli (e.g., *E. coli, Enterobacter cloacae*, and *K. pneumoniae*) for which omadacycline can offer monotherapy for many polymicrobial wound infections. As an illustration, a pooled *post-hoc* analysis of the clinical trials for skin and soft tissue infections in diabetes at post-therapy evaluation found clinical success in the diabetic group in 33/33 (100%) compared to linezolid of 53/61 (87%, a difference that was statistically significant (*P* = 0.047) (61). It is possible that the increase in the spectrum of omadacycline may be an advantage in diabetic patients and others who are more susceptible to skin infections by polymicrobial gram-positive and gram- negative pathogens.

**Fig 2:**
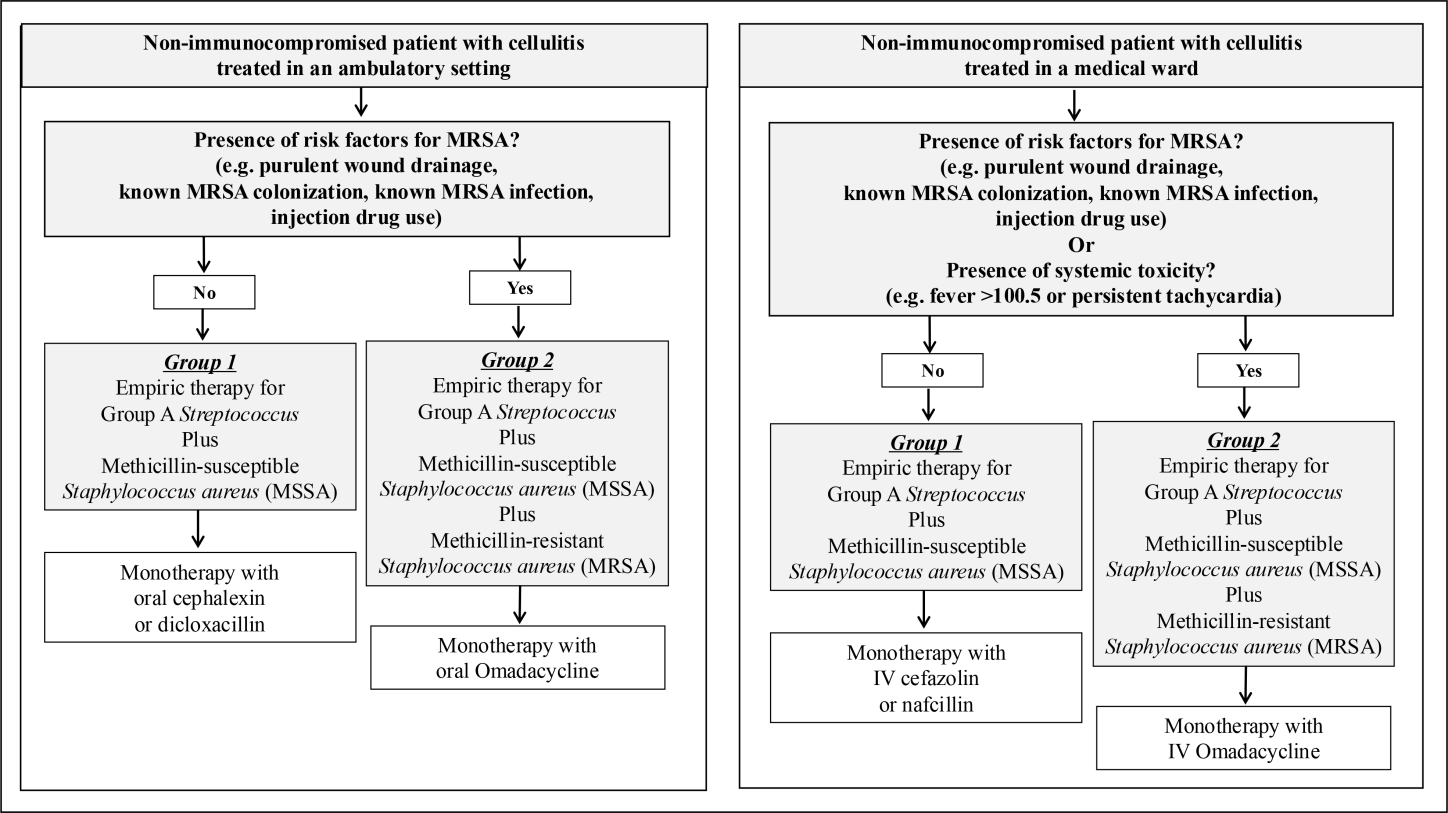
Use of omadacycline for cellulitis.

Our approach to the use of omadacycline for cellulitis is shown in Fig. 2. Empiric antibiotic therapy for cellulitis should cover beta-hemolytic streptococci and MSSA (62). In non-immunocompromised patients with cellulitis, expanded empiric therapy to cover for MRSA is necessary if specific risk factors are present such as purulent wound drainage, known MRSA colonization or infection, or injection drug use. For patients treated in the ambulatory setting without MRSA risk factors, narrow empiric therapy with oral cephalexin or dicloxacillin is appropriate. Patients with risk factors for MRSA (see Fig. 2: ambulatory setting Group 2) can be managed with oral omadacycline starting at a dose of 450 mg for 2 days, followed by 300 mg daily.

In hospitalized non-immunocompromised patients with cellulitis, consideration should be given to the presence of risk factors for MRSA or if the patient presents with systemic signs of toxicity such as fever, sustained tachycardia, or hypotension. Hospitalized patients without risk factors for MRSA or evidence of systemic toxicity can be managed with intravenous cefazolin or nafcillin. Patients with risk factors for MRSA and/or systemic toxicity (see Fig. 2): medical ward Group 2) can be treated with intravenous omadacycline starting with a loading dose of 200 mg on day 1, followed by 100 mg daily. Once patients reach clinical stability, intravenous omadacycline can be switched to oral therapy at a dose of 300 mg daily.

For both CAP and ABSSSI, omadacycline can be an appropriate option for those patients who are either allergic to or intolerant of beta-lactams, macrolides, or fluoroquinolones. For skin infections, omadacycline showed good clinical success compared to linezolid, an agent that may be difficult to use in light of significant drug interactions—especially if patients are taking multiple serotonergic agents. Omadacycline acquisition cost will be a significant barrier to access for many patients. The current average wholesale price of omadacycline maintenance dosing is approximately $600 per day in the United States (63). In contrast, almost all alternative oral therapies for CAP and ABSSI, including linezolid, can be obtained without insurance for around $30 or less for an entire treatment course (64). A patient assistance program is available (www.Nuzyra.com/hcp/affordability).

### Mycobacterium abscessus

Because of the resistance of *M. abscessus* and poor tolerability of patients to many of the antimicrobials used to treat this pathogen, there is a real need for better options of therapy. Both the *in vitro* and *in vivo* evidence suggests omadacycline has potency against *M. abscessus* and the other rapid grower NTMs. Real-world clinical experience indicates that omadacycline as part of combination therapy can now be considered a first-line agent for treatment (65). The availability of both IV and oral administration presents an advancement over many other agents. To date, it appears reasonably well tolerated in long-term use for these infections.

## CONCLUSION

Omadacycline offers advantages over prior tetracycline agents for CAP, ABSSSI, and NTM. It can be positioned for monotherapy for many CAP and ABSSSI (Fig. 1 and 2). The availability of both IV and oral formulation offers flexibility regarding the route of administration and for feasible switch from IV to oral therapy. Once-daily administration is convenient and improves adherence. That there is no need to dose adjust for reduced renal function is an advantage to be consideration in patients who may have azotemia or rapidly changing renal function. Because of the effect that food and multivalent cation-containing medications have on absorption, education on administration and screening for drug–drug interactions, including over-the-counter medications, is important with oral tetracyclines including omadacycline. While omadacycline is a branded antimicrobial, its cost will be higher than those of generic agents; however, this can be justified on the basis of clinical efficacy and safety. Thus, for the general practitioner, omadacycline offers a useful new effective option of therapy for CAP or SSSTI; and for providers who treat NTM (e.g., pulmonary or infectious diseases specialists), omadacycline offers an effective agent for first-line therapy as part of a combination regimen.

## AFTER EPUB

*[This article was published on 16 January 2025 with an error in Fig. 1. The figure was corrected in the current version, posted on 24 January 2025.]*
